# Sugar and stroke in the Western Pacific region—Starting healthy habits early

**DOI:** 10.1016/j.lanwpc.2022.100657

**Published:** 2022-11-30

**Authors:** The Lancet Regional Health – Western Pacific

Stroke is the second leading cause of death worldwide. It is a medical emergency requiring immediate treatment. A stroke occurs when a blood vessel delivering essential nutrients and oxygen to the brain is blocked or ruptures. Brain cells start to die at a rate of about 2 million per minute during a stroke. While the brain makes up only 2% of the total bodyweight, it consumes 20% of the body's energy, derived primarily from blood glucose or sugar. Sadly, many people do not receive life-saving treatment, making stroke a major cause of death and disability across the Western Pacific region, particularly in East Asia. Diabetes is a major modifiable risk factor for stroke. The link between diabetes and stroke is primarily because over time, high blood sugar damages the blood vessels, which can lead to heart disease, and thereby increase the risk of stroke. People with diabetes have about twice the risk of stroke compared with people without diabetes. An estimated 16% of people with diabetes die from stroke, making it one of the leading causes of death and disability in this group.

Most stroke disability-adjusted life-years are attributable to metabolic risks such as high systolic blood pressure and high body-mass index (72%), followed by behavioural risk factors such as an unhealthy diet and physical inactivity (66%). LaMonica and colleagues reported that there was more than a two-fold higher prevalence of diabetes and hypertension in young Samoan adults compared with estimates from other lower-income and middle-income countries. Worryingly, most Samoans presented with prediabetes (rather than diabetes), whereby blood sugar levels are higher than normal but not yet high enough to be diagnosed as diabetes. These findings align with a retrospective study of more than 1 million people from Hong Kong, which showed that prediabetes was associated with greater morbidity and mortality, especially in younger age groups, even after adjusting for metabolic factors and excluding people who developed diabetes. In China, the prevalence of stroke is highest in the northern and eastern regions, potentially linked to unhealthier diets and lack of access to stroke care. Similarly, non-Europeans, especially Māori in New Zealand, were more likely to have reduced access to acute stroke units, brain imaging, and post-stroke interventions, despite having more stroke risk factors. As such, effective blood sugar control through healthy lifestyle changes is an essential component of stroke prevention and reducing disparities in stroke outcomes.

Modifiable risk factors commonly associated with non-communicable diseases such as stroke and diabetes often emerge early in life. Approximately 70% of premature deaths in adulthood are estimated to be due to health-related behaviours formed in childhood and adolescence. In particular, physical inactivity and unhealthy eating habits, especially excess consumption of added sugars in young people, given its association with poor nutritional quality, obesity, and increased risk of diabetes, heart disease, and stroke in later life. In the Western Pacific region, an estimated 7·2 million children younger than 5 years were overweight or obese in 2018, and 84 million children aged 5–19 years were overweight or obese, reflecting a 46% increase from 2010 to 2016. In Australia, nearly 95% of children do not meet the recommended daily intake of vegetables, and Australian companies exporting sugar-sweetened drinks to the Pacific Islands at the forefront of an obesity epidemic have been heavily criticised. In addition to an unhealthy diet, smoking, and alcohol use, lack of physical activity among youth is a major contributor to the increasing burden of non-communicable diseases in Viet Nam, accounting for 11% of deaths.

A greater shift in mentality towards child health to reduce the future burden of stroke and diabetes is urgently needed to ensure lifelong health behaviour change. Successful stroke prevention strategies are often hampered by a lack of public awareness and knowledge about stroke and associated modifiable risk factors. A life-course approach to optimise the effectiveness of stroke prevention strategies at all ages is paramount. In addition to creating an environment that promotes the adoption and maintenance of healthy habits, governments, in collaboration with a broad range of stakeholders, need to place greater focus on combatting the prevalence of modifiable risk factors for stroke and diabetes among children and young people. Specific strategies could include clinicians placing greater emphasis on the benefits of a healthy lifestyle for one's children as part of primary prevention efforts. At the individual level, we can also teach and model healthy habits for our children when possible, through simple behaviours, such as sitting together at mealtimes with minimal distractions, or more complex tasks, such as food shopping and cooking to expose children to fruits and vegetables. Promoting healthy habits in our children starts with us!

